# Supplementary material for: Knowledge, Vaccination Status, and Reasons for Avoiding Vaccinations against Hepatitis B in Developing Countries: A Systematic Review
Source: Vaccines (Basel). 2021 Jun 9;9(6):625. doi: 10.3390/vaccines9060625 (PMC8227242; doi:10.3390/vaccines9060625)
Supplement: Supplementary file 1 [file vaccines-09-00625-s001.zip › Suplementary 2.pdf]

Table S2. Keyword statements and search strategies.

Set#	Searched for
**S1**	Search:(MESH.EXACT.EXPLODE("Knowledge, Attitudes, Practice") OR (MESH.EXACT.EXPLODE("Knowledge") OR (MESH.EXACT.EXPLODE("Health attitudes") (MESH.EXACT.EXPLODE("KAP"))))
**S2**	Search: (MESH.EXACT.EXPLODE("awarenesses") OR (MESH.EXACT.EXPLODE ("awareness") OR(MESH.EXACT.EXPLODE("Vaccination awareness") OR (MESH.EXACT.EXPLODE("Knowledge, Attitudes, Practice"))))
**S3**	Search: (ti,ab("know" or "aware" or "attitude" or "perception" or "belief" or "accept" or "practice" or "kap" or "KAP"))
**S4**	**S3 OR S2 OR S1**
**S5**	Search: (MESH.EXACT.EXPLODE("Immunization Program") OR (MESH.EXACT.EXPLODE("Immunization") OR (MESH.EXACT.EXPLODE("Immunisation") OR (MESH.EXACT.EXPLODE("Vaccination") OR (MESH.EXACT.EXPLODE("Campaign") OR (MESH.EXACT.EXPLODE("Vaccination Promotion")
**S6**	Search: (MESH.EXACT.EXPLODE("Health Personnel") OR MESH.EXACT.EXPLODE ("Healthcare Worker") OR MESH.EXACT.EXPLODE ("Healthcare Provider") OR MESH.EXACT.EXPLODE ("Paramedic") OR MESH.EXACT.EXPLODE ("Adult") OR MESH.EXACT.EXPLODE ("Student") OR MESH.EXACT.EXPLODE ("Patient"))))))
**S7**	Search: (MESH.EXACT.EXPLODE("Hepatitis B") OR (MESH.EXACT.EXPLODE("Hepatitis B vaccine") OR (MESH.EXACT.EXPLODE("Hepatitis B not human papilloma virus ") OR (MESH.EXACT.EXPLODE("Hepatitis B not influenza ")
**S8**	**S7 AND S6 AND S5**
**S9**	**S8 AND S4**
**S10**	Filter: (S9) and (human(yes)) and (la.exact("English")) and (la.exact("Indonesian")) and (la.exact("the last 10 years"))
