# Supplementary material for: Knowledge, Vaccination Status, and Reasons for Avoiding Vaccinations against Hepatitis B in Developing Countries: A Systematic Review
Source: Vaccines (Basel). 2021 Jun 9;9(6):625. doi: 10.3390/vaccines9060625 (PMC8227242; doi:10.3390/vaccines9060625)
Supplement: Supplementary file 1 [file vaccines-09-00625-s001.zip › Suplementary 3.pdf]

Table S3.1. Evidence quality based on the Newcastle-Ottawa Scale (NOS) adapted for cross-sectional study

		Selection					Comparability	Outcome			Total (max.11)	
**No**	**Study**	**Representative-ness of the exposed**	**Adequacy of participant rate**	**Sample size**	**Non-respondents**	**Ascertainment of the exposure (risk factors)**	**Comparability of subjects**	**Assessment of outcome**	**Statistical test (knowledge)**	**Statistical test (vaccination status)**	**Knowledge**	**Vaccine status**
1	Aaron D, 2017	0	1	1	0	0	2	0	0	1	N/A	**5**
2	Abeje G, 2015	1	0	1	0	0	0	0	0	0	**2**	**2**
3	Abiodun, 2019	0	1	0	1	0	0	1	0	0	**3**	**3**
4	Abiola, 2016	1	1	1	1	0	0	1	0	0	**5**	**5**
5	Abiola, 2013	0	1	1	1	0	0	1	0	0	**4**	**4**
6	Adenkanle, 2014	1	0	1	1	0	2	1	1	1	N/A	**7**
7	Adenlewo, 2017	0	1	0	0	0	0	0	0	0	**1**	**1**
8	Adeyemi, 2013	0	0	1	1	0	2	1	1	1	**6**	**6**
9	Adjei, 2018	0	1	1	1	0	2	1	0	0	**6**	N/A
10	Ahmad, 2016	1	0	1	0	0	0	1	0	0	**3**	**3**
11	Akibu, 2018	1	1	1	0	0	2	1	0	1	N/A	**7**
12	Al-Hazmi, 2019	0	1	0	0	0	0	1	0	0	**2**	**2**
13	Alavian, 2011	0	1	0	0	0	0	0	0	0	**1**	N/A
14	Alese, 2016	0	0	0	0	0	0	0	0	0	N/A	**0**
15	Ali, 2017	1	1	0	0	0	0	0	0	0	**2**	N/A
16	Alqahtani, 2014	1	1	1	1	0	0	0	0	0	**4**	N/A
17	Aniaku, 2019	1	0	1	1	0	0	0	0	0	**3**	**3**

18	Aroke, 2018	0	1	0	0	0	0	1	0	0	**2**	**2**
19	Asif, 2011	0	1	0	0	0	0	1	0	0	N/A	**2**
20	Assuncao, 2012	1	1	1	1	0	2	0	0	1	N/A	**7**
21	Attaullah, 2011	1	0	0	0	0	0	0	0	0	N/A	**1**
22	AydemİR, 2016	0	1	0	0	0	0	0	0	0	N/A	**1**
23	Bedaso, 2018	1	1	1	0	0	0	1	0	0	**4**	**4**
24	Bekele, 2014	0	0	0	0	0	0	1	0	0	N/A	**1**
25	Celikel, 2014	0	1	0	0	0	0	1	0	0	N/A	**2**
26	Chan, 2011	0	1	0	0	0	2	0	1	0	**4**	N/A
27	Chao, 2010	0	0	0	0	0	2	0	1	0	**3**	N/A
28	Chingle, 2017	1	1	0	1	0	0	0	0	1	N/A	**4**
29	Choudhary, 2017	0	0	0	0	0	0	0	0	0	**0**	**0**
30	Chung, 2012	0	1	0	0	0	2	1	0	0	**4**	**4**
31	da Costa, 2013	1	1	0	0	0	2	1	0	1	N/A	**6**
32	de Souza, 2014	0	0	0	0	0	0	0	0	0	N/A	**0**
33	Debes, 2016	0	1	0	0	0	0	0	0	0	**1**	**1**
34	Demsiss, 2018	0	1	1	1	0	2	1	0		**6**	N/A
35	Dev, 2018	0	0	1	1	0	0	1	0	0	**3**	**3**
36	Eni, 2019	0	1	0	0	0	2	0	1	0	**4**	**3**
37	Ferreira, 2012	0	1	0	0	0	2	1	0	1	N/A	**5**
38	Ghomraoui, 2016	1	1	1	0	0	0	0	1	1	**4**	**4**
39	Guerra, 2018	0	0	1	1	0	0	0	0	0	N/A	**2**
40	Hebo, 2019	1	1	1	0	0	0	1	0	0	**4**	N/A

41	Ibrahim, 2014	1	0	0	0	0	0	0	0	0	**1**	**1**
42	Iqbal, 2019	0	0	0	0	0	0	0	0	0	N/A	**0**
43	Jaquet, 2017	1	0	0	0	0	2	0	1	0	**4**	N/A
44	Joukar, 2018	1	0	0	0	0	2	1	0	0	**4**	N/A
45	Kesieme, 2011	1	0	0	0	0	0	0	0	0	**1**	**1**
46	Khan, 2010	0	0	0	0	0	0	1	0	0	**1**	**1**
47	Khandelwal, 2018	1	0	0	0	0	0	1	0	0	**2**	**2**
48	Ko, 2017	0	0	0	0	0	2	1	0	1	N/A	**4**
49	Kouassi, 2017	1	0	0	0	0	2	0	0	1	N/A	**4**
50	Lee, 2010	0	0	0	0	0	2	1	1	0	**4**	**3**
51	Li, 2015	0	1	0	0	0	0	1	0	0	**2**	N/A
52	Machiya, 2015	1	0	1	0	0	2	0	0	1	**4**	**5**
53	Meriki, 2018	0	0	1	1	0	2	1	0	0	N/A	**5**
54	Mirzaei, 2019	1	1	1	0	0	2	1	0	1	N/A	**7**
55	Moezzi, 2016	0	0	1	0	0	0	0	0	0	N/A	**1**
56	Mungandi, 2017	1	0	0	0	0	2	0	0	1	**3**	**4**
57	Mursy, 2019	1	0	0	0	0	0	1	0	0	**2**	**2**
58	Mustafa, 2018	1	0	1	0	0	0	0	0	0	**2**	**2**
59	Mustufa, 2010	1	0	0	0	0	0	1	0	0	N/A	**2**
60	Noreen, 2015	1	1	1	1	0	0	1	1		**6**	N/A
61	Noubiap, 2013	0	0	0	0	0	0	1	0	0	**1**	**1**
62	Noubiap, 2014	0	0	0	0	0	0	1	0	1	**1**	**2**
63	Ogoina, 2014	0	0	0	0	0	2	0	0	1	N/A	**3**

64	Okwara, 2012	0	1	0	1	0	0	0	1	1	**3**	**3**
65	Omotowo, 2018	0	1	0	0	0	2	0	0	1	**3**	**4**
66	Oyewusi, 2015	1	1	0	0	0	0	0	0	0	**2**	**2**
67	Park, 2012	1	0	0	0	0	2	0	0	1	N/A	**4**
68	Park, 2013	1	0	0	0	0	2	0	0	1	N/A	**4**
69	Pathoumthong, 2014	1	1	0	0	0	2	0	0	1	**4**	**5**
70	Rajamoorthy, 2019	1	1	1	1	0	2	1	1	0	**8**	N/A
71	Rathi, 2018	0	1	0	0	0	0	1	0	0	**2**	N/A
72	Ray, 2017	0	0	0	0	0	0	0	0	0	**0**	**0**
73	Resende, 2010	1	1	1	0	0	2	1	0	1	N/A	**7**
74	Roushan, 2013	1	1	0	0	0	2	1	1	0	**6**	N/A
75	Sandeep, 2010	1	1	0	0	0	0	1	0	0	**3**	N/A
76	Shahbaz, 2014	1	0	0	0	0	0	0	0	0	**1**	**1**
77	Shakeel, 2015	0	0	0	0	0	0	1	0	0	**1**	**1**
78	Shukla, 2016	1	0	0	0	0	0	1	0	0	**2**	**2**
79	Singh, 2011	0	1	0	0	0	0	1	0	0	**2**	**2**
80	Tatsilong, 2016	0	0	1	1	1	2	1	1	0	**7**	**6**
81	Usmani, 2010	1	0	0	0	0	0	1	0	0	N/A	**2**
82	Vo (5), 2018	1	1	1	1	0	0	1	0	0	**5**	**5**
83	Vo (8), 2018	1	0	1	1	0	2	1	1	0	**7**	N/A
84	Yamazhan, 2011	0	1	1	0	0	2	1	0	0	**5**	**5**
85	Yang, 2015	1	0	0	0	0	0	1	0	0	**2**	N/A
86	Yuan, 2019	0	1	0	0	0	2	0	0	1	N/A	**4**

**Total**	**42**	**43**	**30**	**20**	**1**	**68**	**47**	**14**	**23**	**182**	**207**

Table S3.2. Evidence quality based on the Newcastle-Ottawa Scale (NOS) adapted for cohort study

	**Selection**				**Comparability**		**Outcome**			**Total (max. 9)**	
**Study**	**Representative-ness of the exposed**	**Selection of the non exposed cohort**	**Ascertainment of exposure**	**Demonstration that outcome of interest was not present at start of study**	**Comparability of cohort**	**Study controls for at least 3 additional risk factors**	**Assessment of outcome**	**Follow-up long enough for outcomes to occure**	**Adequacy of follow up of cohorts**	**Knowledge**	**Vaccine status**
Zheng, 2017	1	1	1	1	1	1	1	1	1	**N/A**	**8**

Table S3.3. Evidence quality based on the Newcastle-Ottawa Scale (NOS) adapted for case-control study

	**Selection**				**Comparability**		**Exposure**			**Total (Max. 9)**	
**Study**	**Case definition adequte**	**Representative-ness of the cases**	**Selection of controls**	**Definition of control**	**Comparability of cases and control**	**Study controls for at least 3 additional risk factors**	**Ascertainment of exposure**	**Same method cases and controls**	**Non-response rate**	**Knowledge**	**Vaccine status**
Zafrin, 2017	1	1	1	1	1	1	1	1	1	**N/A**	**8**
